# Respiratory–Swallow Training in Head and Neck Cancer: Impact on Swallowing Safety and Efficiency

**DOI:** 10.1044/2026_JSLHR-26-00075

**Published:** 2026-08-31

**Authors:** Bonnie Martin-Harris, Kate Davidson, David McFarland

**Affiliations:** a Roxelyn and Richard Pepper Department of Communication Sciences and Disorders, School of Communication, Northwestern University, Evanston, IL; b Otolaryngology—Head and Neck Surgery, Radiation Oncology, Feinberg School of Medicine, Northwestern University, Chicago, IL; c Division of Speech-Language Pathology, Department of Rehabilitation Sciences, College of Health Professions, Medical University of South Carolina, Charleston; d École d'orthophonie et d'audiologie, Faculté de médecine, Université de Montréal, Quebec, Canada; e Centre de recherche du Centre hospitalier universitaire Sainte-Justine, Montréal, Quebec, Canada; f Centre for Research on Brain, Language and Music, McGill University, Montréal, Quebec, Canada

## Abstract

**Purpose:**

This review article provides a programmatic translational synthesis of a research program investigating respiratory–swallow coordination as a modifiable physiologic target underlying swallowing impairment and safety, with a focus on individuals treated for head and neck cancer (HNC). The purpose is to synthesize physiologic evidence, clinical trial outcomes, and translational advances supporting respiratory–swallow training (RST) as a mechanism-based intervention.

**Method:**

Evidence is drawn from a series of experimental, observational, and interventional studies conducted over more than 3 decades. Methods include synchronized videofluoroscopy and respiratory measurement to characterize respiratory–swallow coordination, clinical trials evaluating RST across chronic and earlier recovery-stage HNC populations, and ongoing work incorporating wearable sensor technology and synchronous telehealth delivery to support ambulatory monitoring and home-based training. Data from Phase I, Phase II, and ongoing randomized efficacy trials are summarized to illustrate mechanistic and clinical effects of RST.

**Results:**

Across studies, swallowing was most safely and efficiently initiated during mid- to low-quiet (tidal) breathing expiratory lung volumes, whereas deviations from this pattern were associated with increased airway invasion and physiologic impairment. RST reliably increased expiratory-phase swallow initiation and was associated with improvements in swallowing impairment, airway protection, bolus clearance, functional oral intake, and patient-reported outcomes. Phases I and IIa trial data demonstrated significant reductions in physiologic impairment scores and penetration–aspiration severity following training.

**Conclusions:**

Collectively, these findings support respiratory–swallow coordination as a modifiable target for dysphagia rehabilitation that results in important physiologic and airway protection advantages. RST represents a mechanism-driven approach that extends beyond compensatory strategies and shows promise for scalable, technology-enabled delivery in both clinical and ambulatory settings. This work has implications for swallowing assessment, intervention, and future research across clinical populations.

Swallowing is a complex, highly coordinated sensorimotor process that is tightly integrated with respiration (Martin-Harris, 2006; Martin-Harris et al., 2023). Under typical conditions, swallowing occurs within a narrow and physiologically advantageous window of the respiratory cycle, most often during expiration and at mid- to low-quiet (tidal) breathing lung volumes (Martin-Harris et al., 2005). This coordination is not incidental; rather, it reflects an evolved cross-system interaction that supports airway protection, efficient bolus clearance, and rapid resumption of breathing following the swallow (Martin-Harris, 2006; McFarland et al., 2018). Disruption of respiratory–swallow coordination has direct clinical consequences, contributing to swallowing impairment, airway invasion, and downstream impacts on health, nutrition, and quality of life (Hopkins-Rossabi et al., 2021; Yoshida et al., 2024).

Individuals treated for oropharyngeal head and neck cancer (HNC) represent a particularly vulnerable population in which to study the consequences of disrupted breathing–swallowing coordination. Multimodality cancer treatment, including surgery, radiation, and chemotherapy, can alter sensory input, motor execution, tissue compliance, and neural control across the aerodigestive tract (Hutcheson et al., 2017; King et al., 2016). These changes often persist well beyond the acute phase of treatment and contribute to refractory and chronic dysphagia characterized by excessive oropharyngeal residue, aspiration, and reduced quality of life (Miller et al., 2025; Sellstrom et al., 2025). Importantly, swallowing impairment in this population cannot be fully explained by isolated structural or biomechanical deficits alone, suggesting that higher level coordination across physiologic systems may play a critical role.

For more than 3 decades, our research programs have focused on defining the physiologic principles governing respiratory–swallow coordination, identifying how these principles are disrupted in clinical populations, and developing mechanism-driven interventions to restore more optimal patterns of coordination (Martin-Harris et al., 2023). Early foundational work established the temporal and biomechanical relationships between respiratory phase, lung volume, and key swallowing events using respiratory kinematics, airflow measures, and videofluoroscopy (Martin, 1991; Martin-Harris et al., 2005; McFarland et al., 1994; McFarland & Lund, 1995). Subsequent studies demonstrated that deviations from typical expiratory-phase swallow initiation are associated with increased swallowing impairment and airway invasion, particularly in individuals treated for HNC (Brodsky et al., 2010). More recent work has further shown that disrupted respiratory–swallow coordination is systematically related to the severity of swallowing impairment in this population (Hopkins-Rossabi et al., 2021).

The results of these studies served as a platform for the development and testing of respiratory–swallow training (RST; Martin-Harris et al., 2015, 2023), a targeted, skill-based intervention designed to retrain swallow initiation to occur during expiration. RST is grounded in principles of experience-dependent neuroplasticity and motor learning, in which repeated, task-specific practice drives adaptive changes in neural control and motor behavior (Huckabee et al., 2023; Kleim & Jones, 2008; Robbins et al., 2008). In contrast to compensatory or strength-based approaches that modify bolus flow or target peripheral musculature, this approach emphasizes restoration of precise motor control through training of timing, coordination, and task execution. RST emphasizes identification, acquisition, and mastery of appropriate respiratory–swallow timing as a skill-based intervention rather than a compensatory or task-specific within-swallowing-system strategy. Across a series of clinical trials in refractory and chronic HNC populations, RST has been shown to improve respiratory–swallow coordination, swallowing physiology, airway protection, and selected functional and patient-reported outcomes (Graham et al., 2026; Martin-Harris, 2025; Martin-Harris et al., 2015).

More recently, advances in wearable sensor technology and remote data acquisition have enabled studies extending this work beyond laboratory-based assessments to ambulatory, real-world contexts (Kang et al., 2022; Martin-Harris et al., 2023). These technological innovations facilitate continuous monitoring of breathing and swallowing patterns, support telehealth delivery of RST, and offer new opportunities to examine the generalization and durability of training effects.

The purpose of this review article is to provide a programmatic translational synthesis of the research trajectory that has established respiratory–swallow coordination as a target for dysphagia rehabilitation in HNC at a critical stage of clinical and technological advancement. Rather than offering a comprehensive review of the broader swallowing literature, this narrative synthesis is organized around milestone physiologic, observational, interventional, and translational studies from this line of work. In contrast to prior work that emphasized broader cross-system integration of respiration and deglutition (Martin-Harris et al., 2023), this review article focuses specifically on the translational arc of RST in HNC, including disorder characterization, intervention development, clinical trial evidence, and ambulatory translation, defined here as the extension of assessment and intervention beyond laboratory and clinic settings into real-world environments through wearable sensors, remote monitoring, and telehealth-enabled delivery. In doing so, it serves as a roadmap from foundational discovery to intervention development, clinical trial evaluation, and ambulatory translation while distinguishing well-established findings from those that remain emerging and identifying next-phase research priorities.

## Physiologic Basis of Respiratory–Swallow Coordination

Swallowing is normally superimposed on spontaneous, quiet breathing and requires a brief, well-timed interruption of the respiratory cycle (Kijima et al., 1999; McFarland et al., 2018). This interruption is not random; rather, swallowing initiation is constrained to a relatively narrow range of respiratory phase and lung volume that optimizes airway protection and bolus clearance while minimizing disruption to ventilation (McFarland et al., 2018). Experimental and observational studies using synchronized measures of respiration and swallowing have consistently shown that, in healthy adults, swallows are most frequently initiated during expiration and at mid- to low-lung volumes within the quiet (tidal) breathing cycle (Martin, 1991; Martin-Harris et al., 2005; McFarland et al., 2016).

From a biomechanical perspective, the expiratory phase of respiration provides a favorable set point for the coordinated movements required for safe and efficient swallowing (see Figure 1). During expiration, the tongue base assumes a more posterior position, the pharyngeal lumen is relatively narrowed, larynx is positioned superiorly, and the arytenoid-true vocal fold complex assumes a paramedian location (Martin-Harris, 2006; McFarland et al., 2018). These conditions facilitate timely laryngeal vestibular closure and tongue base retraction necessary for airway protection and efficient pharyngeal clearance during swallowing (Martin-Harris et al., 2023). In contrast, swallow initiation during inspiration is associated with forward tongue positioning, pharyngeal widening, caudal displacement of the larynx, and vocal fold abduction, creating counterproductive traction on structures critical for airway protection and bolus propulsion (Martin-Harris et al., 2005; McFarland et al., 2016).

**Figure 1.**
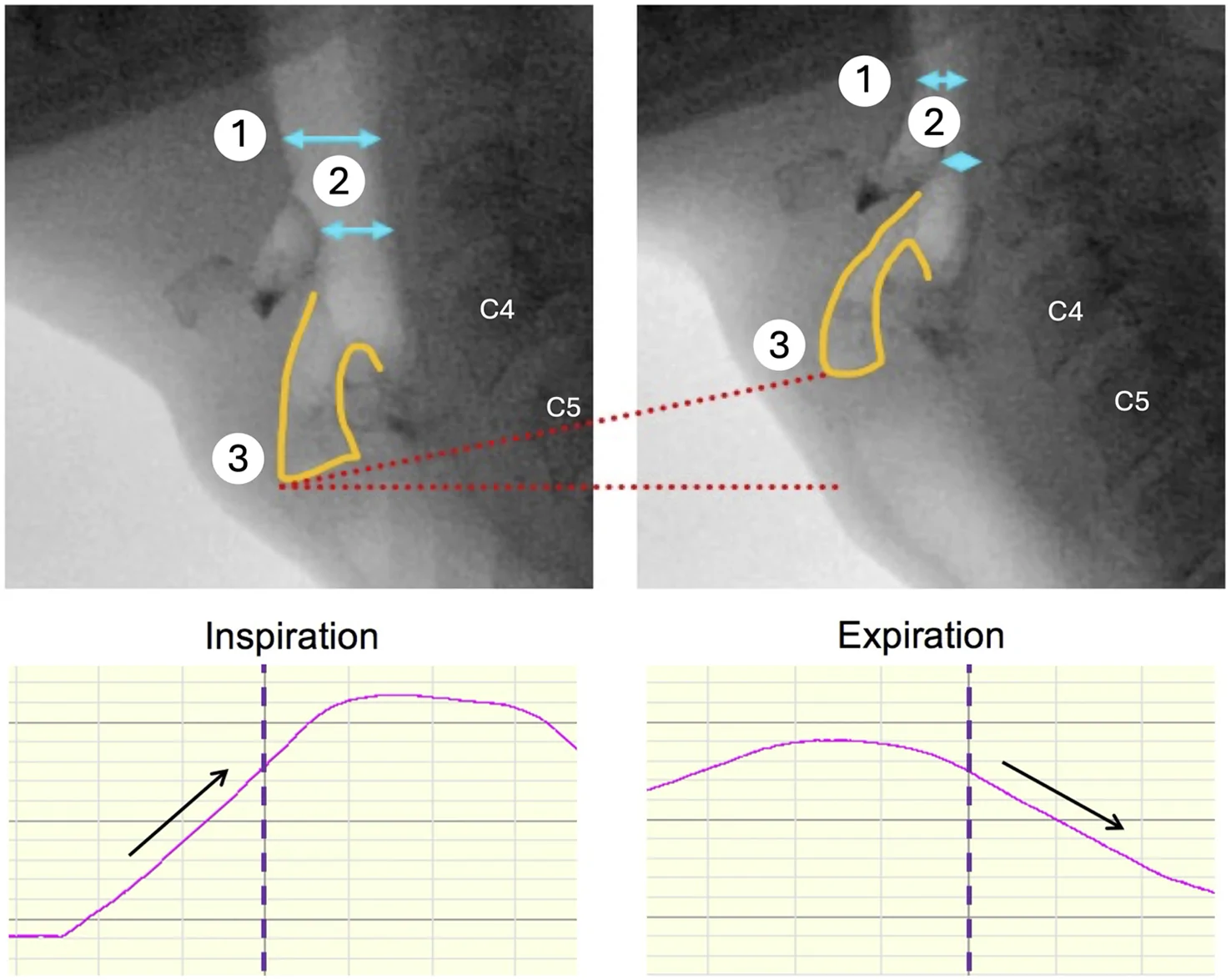
(1) Tongue base, (2) pharyngeal lumen, and (3) larynx positioning during inspiration (left) and expiration (right), highlighting biomechanical conditions relevant to swallow initiation and airway protection. C4 = 4th cervical vertebrae; C5 = 5th cervical vertebrae.

Lung volume has potential to further constrain this coordination (McFarland et al., 2016, 2018). Swallow initiation typically occurs at lower quiet (tidal) breathing lung volumes during expiration, when the diaphragm is returning toward its resting position and resistance to laryngeal elevation is minimized. At these volumes, partial adduction of the vocal folds and inward posturing of the arytenoid-vocal fold complex create a mechanical advantage for rapid and complete airway closure. Additionally, relaxation of the crural diaphragm during expiration may facilitate downstream bolus transport by reducing resistance at the lower esophageal sphincter, underscoring the integrated nature of respiratory and swallowing mechanics across the aerodigestive tract (Martin-Harris et al., 2005; McFarland et al., 2016; Mittal et al., 1995).

Temporal analyses of swallowing events relative to respiratory activity further support the existence of tightly coupled breathing and swallowing. Using synchronized videofluoroscopy and respiratory measures, prior work (Martin-Harris et al., 2005) has demonstrated consistent temporal relationships among key swallowing events, including laryngeal vestibular closure, peak hyoid excursion, pharyngoesophageal segment opening, and respiratory cessation onset and offset that are preserved when swallowing occurs within the optimal expiratory window (see Figure 2). These functional relationships are consistent with coordinated brainstem patterning that temporarily resets the respiratory rhythm to accommodate swallowing and then facilitates timely resumption of breathing following bolus passage from the laryngeal airway (Martin, 1991; Martin-Harris et al., 2005; McFarland et al., 2016).

**Figure 2.**
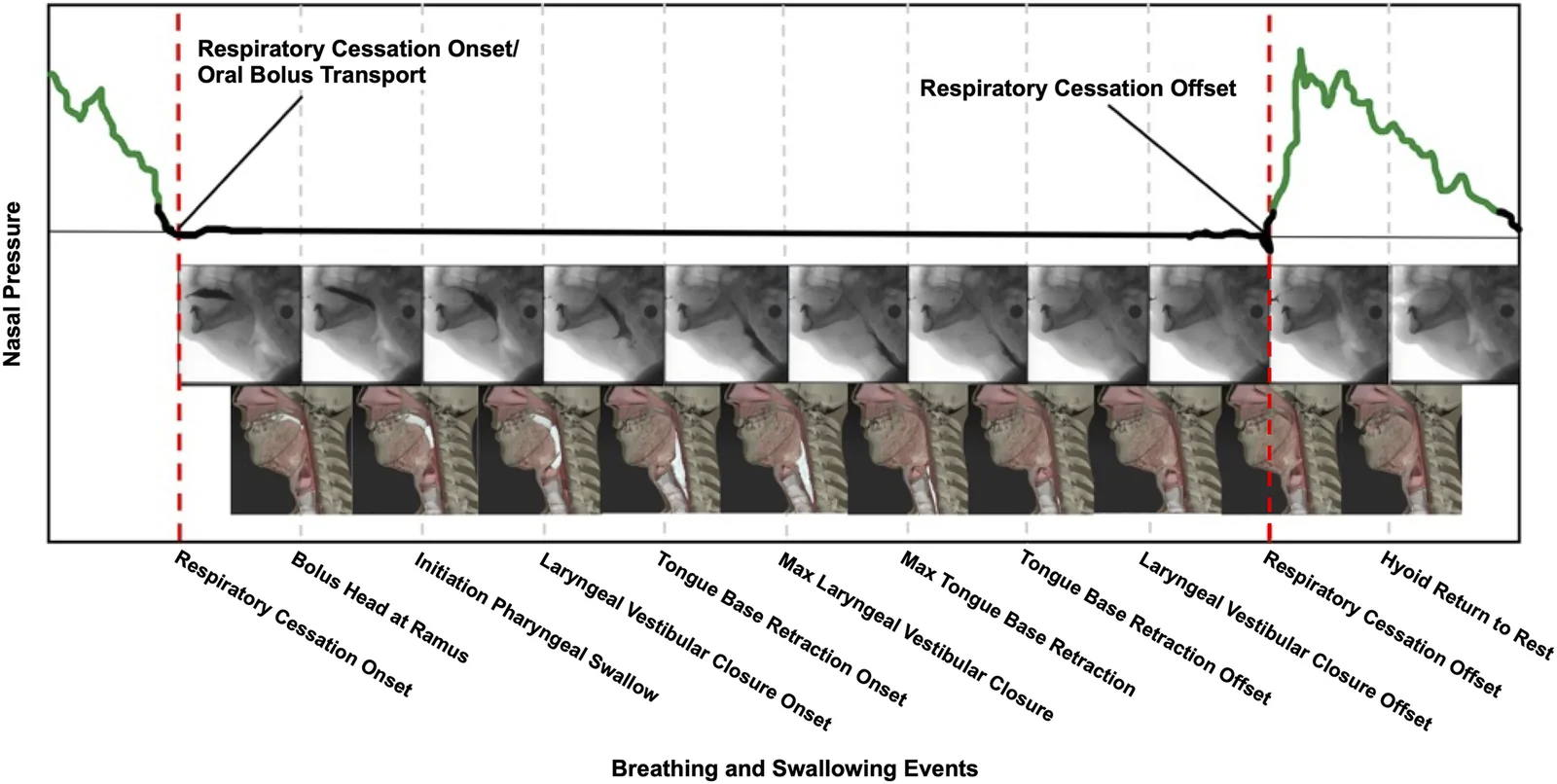
Temporal coordination between breathing and key swallowing events during expiratory-phase swallow initiation.

When swallow duration is prolonged or when swallow initiation occurs outside the typical expiratory window, this coordination becomes less efficient. Atypical swallowing patterns, including delayed swallow initiation and prolonged respiratory pauses, are associated with sensations of air hunger (i.e., a mismatch between respiratory drive and insufficient mechanical lung expansion), altered timing of airway closure, and increased vulnerability to airway invasion (Brodsky et al., 2010; Martin-Harris, 2008). These effects are magnified in clinical populations with compromised respiratory reserve or altered sensory feedback, highlighting the importance of precise timing and coordination across systems rather than isolated movement adequacy (Rangwala et al., 2026; Yoshimatsu et al., 2020).

Collectively, these physiologic data support the central premise of this research program: The respiratory phase and lung volume at which swallowing is initiated influence the biomechanics and safety of the swallow. Optimal coordination reflects an interaction between peripheral sensory feedback, central neural control, and biomechanical constraints of the respiratory and swallowing systems (Martin-Harris et al., 2023). Disruption at any level, sensory, motor, or integrative, can shift swallow initiation outside this optimal window, setting the stage for impaired airway protection and inefficient bolus clearance. This mechanistic framework provides the foundation for targeted intervention strategies aimed at restoring more typical respiratory–swallow coordination rather than compensating for and focusing on swallowing impairments alone. These tightly coordinated respiratory–swallow relationships provide the physiologic basis for efficient airway protection and bolus clearance and establish a framework for understanding how disruption of this coordination contributes to dysphagia.

## Disordered Respiratory–Swallow Coordination in HNC

Disruption of respiratory–swallow coordination is a prominent and clinically meaningful feature of dysphagia in individuals treated for oropharyngeal HNC. Unlike healthy adults, who overwhelmingly initiate swallows during expiration, patients treated for HNC exhibit greater variability in swallow–breathing phase relationships, including an increased frequency of swallows initiated during inspiration or followed immediately by inspiration (Brodsky et al., 2010; Hopkins-Rossabi et al., 2021). These atypical patterns reflect altered cross-system coordination rather than isolated deficits in structural movement or strength and are strongly associated with severity of swallowing impairment (see Table 1).

**Table 1.** Disordered respiratory–swallow coordination in head and neck cancer.

Finding	Brodsky et al. (2010)	Hopkins-Rossabi et al. (2021)
Coordination metric	% E-E swallows	% expiratory-phase swallows; % optimal LVSI
Definition of optimal coordination	Swallow bracketed by expiration	Swallow initiated during expiration at ≤ 50% of tidal volume range
Coordination (controls)	72.5% E-E swallows	—
Coordination (HNC)	37.5% E-E swallows	34% optimal LVSI range
Non-optimal coordination patterns	E-I, I-E, I-I	Inspiratory and high-LVSI
Association with airway invasion	Non–E-E ↑ PAS	↓ expiratory swallows ↑ PAS (*p* = .001)
Association with physiologic impairment	Non–E-E ↑ overall impairment	↓ expiratory swallows ↑ pharyngeal and oral impairment (*p* = .002–.036)
Primary contribution	Identifies presence of respiratory–swallow discoordination in treated HNC	Demonstrates graded relationship between coordination and impairment severity in treated HNC

*Note.* E-E = expiratory–expiratory respiratory–swallow pattern; LVSI = lung volume at swallow initiation; HNC = head and neck cancer; E-I = expiratory–inspiratory; I-E = inspiratory–expiratory; I-I = inspiratory–inspiratory; ↑ = higher; PAS = Penetration–Aspiration Scale (Rosenbek et al., 1996); ↓ = lower.

Using synchronized respiratory measures and videofluoroscopy, Brodsky et al. (2010) provided the first detailed characterization of respiratory–swallow phase patterns in individuals treated for oropharyngeal HNC. In that study, a significant proportion of swallows occurred outside the typical expiratory window, and nonexpiratory patterns were associated with higher rates of airway invasion and overall swallowing impairment. Importantly, these aberrant coordination patterns persisted despite the nature of the oncologic treatment and were observed in the absence of gross anatomic obstruction, underscoring the role of disrupted timing and integration across systems.

Subsequent work extended these findings by demonstrating that respiratory–swallow coordination is not merely altered in individuals treated for HNC but is systematically related to impairment severity. Hopkins-Rossabi et al. (2021) showed that individuals with greater deviation from typical expiratory-phase swallowing exhibited more severe swallowing impairment, supporting respiratory–swallow phase patterning as a meaningful physiologic marker. Together, these data establish disordered respiratory–swallow coordination as a defining feature of dysphagia in this population.

Several mechanisms likely contribute to this disruption following HNC treatment. Radiation and surgery can alter afferent sensory input from the upper aerodigestive tract, degrade the precision of motor execution, and change tissue compliance, all of which may interfere with the central integration required to appropriately time swallow initiation within the respiratory cycle (Caudell et al., 2009; Hutcheson et al., 2017; Logemann et al., 2008). In addition, treatment-related changes in respiratory function, including reduced pulmonary reserve and altered breathing patterns, may further constrain the ability to coordinate swallowing optimally (Brodsky et al., 2010; Martin-Harris, 2008). These factors interact to increase the likelihood that swallow initiation occurs outside the expiratory window, particularly during challenging swallowing tasks such as large-volume liquid swallows or under conditions of fatigue (McFarland et al., 2016).

Critically, disordered respiratory–swallow coordination in individuals treated for HNC has important implications for both airway protection and swallowing efficiency. Inspiratory-phase swallow initiation and postswallow inspiration increase the risk that residual material will be drawn toward the airway, contributing to airway invasion and potential pulmonary complications (Brodsky et al., 2010; Hopkins-Rossabi et al., 2021; Yoshida et al., 2024). At the same time, prolonged or poorly timed respiratory pauses may compromise patient comfort and limit the capacity for repeated swallows during a meal, reducing overall intake and contributing to nutritional compromise (Cassiani et al., 2015; Lin & Shune, 2020). These patterns help explain why dysphagia in HNC often persists despite adequate structural healing and may contribute to broader downstream clinical consequences, including limitations in social participation during eating, malnutrition, and reduced quality of life, and why compensatory strategies and traditional within-swallowing-system therapy alone may be insufficient to address the underlying physiologic deficit.

## Development and Principles of RST

The recognition that disordered respiratory–swallow coordination represents a central physiologic impairment in individuals treated for HNC provided the conceptual foundation for the development of RST and subsequent clinical trials (VA RR&D, C7135R, NCT01032928; VA RR&D, 5I01RX002352, NCT03377270; NIH/NCI, 1R01CA262502, NCT05278039). The RST framework emphasizes restoring a more typical, expiratory-based swallow–breathing pattern to improve swallowing safety and efficiency through skill-based retraining of coordination. RST was designed to target the timing and integration of swallowing within the respiratory cycle as a modifiable physiologic target, reflecting a shift from within-swallowing-system therapy that often focuses on increasing strength and range of swallowing movements to a cross-system rehabilitation intervention grounded in principles of motor and perceptual learning.

RST is predicated on the observation that, even in the presence of chronic structural and sensory changes following cancer treatment, individuals retain the capacity to modify the timing of swallow initiation relative to breathing when provided with explicit feedback and structured practice. Early feasibility work conducted during intervention development demonstrated that HNC patients could reliably identify respiratory phase and adjust swallow initiation accordingly when provided with real-time visual feedback derived from respiratory signals (Martin-Harris et al., 2015). These results supported respiratory–swallow coordination as a learnable skill rather than a fixed physiologic constraint, informing the core design features of RST, which incorporate principles of motor and perceptual learning (Ostry & Gribble, 2016; Vahdat et al., 2014).

The RST protocol is organized around three sequential learning stages: *identification*, *acquisition*, and *mastery*. During *identification*, individuals learn to perceive their own respiratory cycle and recognize the expiratory phase as the optimal window for swallow initiation, under the guidance of speech-language pathologists (SLPs) who support perceptual awareness and interpretation of respiratory signals. This perceptual component is essential, as impaired interoceptive awareness and altered sensory feedback are common following HNC treatment (Mehdizadeh et al., 2020; Riantiningtyas et al., 2024). *Acquisition* focuses on deliberate practice of swallow initiation during expiration under conditions of augmented feedback, with SLPs providing real-time performance cues using respiratory inductance plethysmography and airflow measures during liquid bolus trials (thin, nectar, and honey). *Mastery* emphasizes consistency and generalization, with feedback systematically reduced as individuals demonstrate stable expiratory-phase swallowing and SLPs adjust practice conditions to support transfer across tasks and contexts. Although training is conducted using graduated consistencies of liquid boluses to facilitate controlled mastery of respiratory–swallow coordination, treatment effects have been evaluated using standardized instrumental swallowing assessments that include both liquid and solid bolus trials, demonstrating generalization beyond the trained stimulus (Graham et al., 2026; Martin-Harris, 2025; Martin-Harris et al., 2015).

RST differs from traditional dysphagia interventions in that it does not prescribe a specific swallowing maneuver or postural adjustment. Instead, it targets the temporal coordination between two physiologic systems, respiration and swallowing, allowing the biomechanical benefits of expiratory-phase swallowing to emerge naturally. This distinction is critical, as it positions RST as a skill-based intervention focused on training underlying coordination rather than modifying task performance, and therefore applicable across bolus types and eating contexts rather than a task-specific compensatory strategy.

Building on these foundational principles, more recent work has extended the RST framework through advances in sensing and feedback technology. Automated detection of swallow onset, respiratory phase, and lung volume has enabled objective performance monitoring and real-time feedback with reduced reliance on laboratory instrumentation (Hopkins-Rossabi et al., 2020). Complementary advances in soft, skin-interfaced mechano-acoustic sensors now enable continuous, ambulatory monitoring of respiratory and swallowing biomechanics outside the laboratory, supporting remote assessment, feedback, and training in real-world environments (Kang et al., 2022; see Figure 3). Together, these developments support more efficient training delivery and provide a clear pathway for extending RST beyond specialized clinical settings.

**Figure 3.**
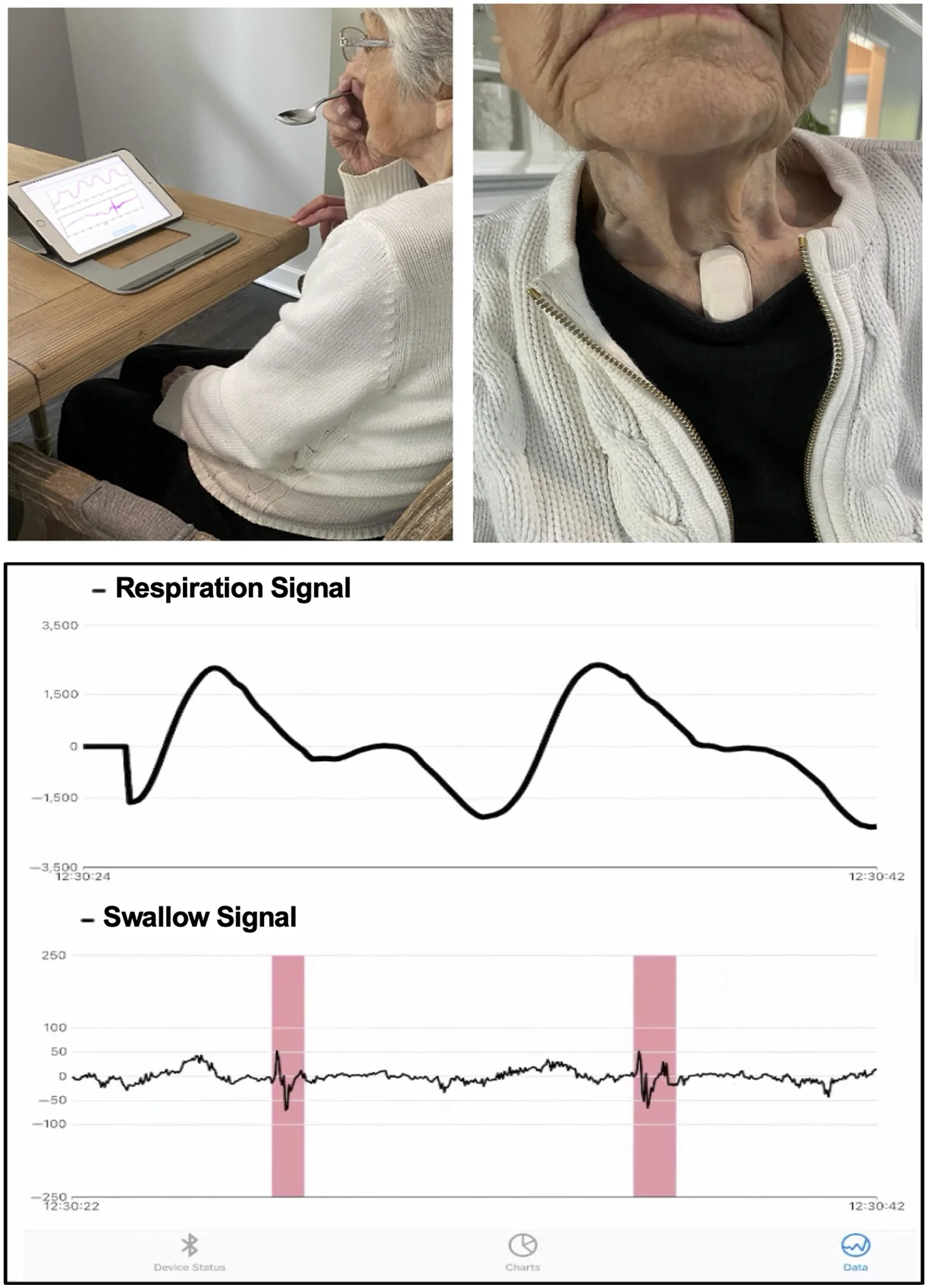
Wearable sensor-based monitoring and remote application–supported telehealth delivery of respiratory–swallow training.

Collectively, these advances position RST within a broader shift toward rehabilitation strategies that target cross-system coordination and control rather than isolated movement components (Martin-Harris et al., 2023). By treating respiratory–swallow coordination as a trainable skill, RST offers a principled framework for addressing dysphagia in populations where structural remediation is limited and highlights the potential for integrating physiologic training with digital health technologies to support durable, real-world behavior change.

## Clinical Trial Evidence and Outcomes of RST

Clinical trials evaluating RST in individuals treated for HNC provide converging evidence that targeting respiratory–swallow coordination yields meaningful improvements in swallowing physiology and airway protection (VA RR&D, C7135R, NCT01032928; VA RR&D, 5I01RX002352, NCT03377270; NIH/NCI, 1R01CA262502, NCT05278039). This evidence base spans early safety and feasibility to randomized efficacy trials, including a published Phase I single-arm, nonrandomized trial (NCT01032928) and a more recent Phase IIa randomized crossover trial (NCT03377270), both conducted in controlled, in-clinic settings using respiratory-inductance plethysmography and nasal airflow for biofeedback. Building on this work, an ongoing Phase IIb randomized, sham-controlled, parallel-group trial (NCT05278039) extends this approach to synchronous telehealth delivery using novel wearable sensor technology for biofeedback, remote monitoring, and ambulatory implementation.

The progression of RST across clinical trial phases, populations, and delivery models is summarized in Table 2, illustrating the evolution of the program from an initial Phase I trial establishing the feasibility and acquisition of respiratory–swallow coordination in individuals with chronic dysphagia following HNC treatment to a Phase IIa randomized efficacy trial in refractory dysphagia and, subsequently, to an ongoing Phase IIb randomized, sham-controlled trial evaluating telehealth delivery, remote monitoring, and ambulatory implementation. Together, these studies reflect a staged translational pathway from physiologic skill acquisition to efficacy testing and real-world application. Across these trials, the primary mechanistic outcome has been an increase in the frequency of swallows initiated during expiration, with associated improvements observed across liquid and solid bolus conditions, confirmed by standardized instrumental swallowing assessments (Graham et al., 2026; Martin-Harris, 2025; Martin-Harris et al., 2015). Changes in this coordination pattern have been accompanied by improvements in swallowing impairment, functional, and patient-reported outcome measures supporting the central role of timing and integration in dysphagia rehabilitation (Graham et al., 2026; Martin-Harris, 2025; Martin-Harris et al., 2015).

**Table 2.** Programmatic progression of respiratory–swallow training.

Study phase	Trial (NCT)	Study design	Population	Intervention delivery	Outcomes	Status
Phase I (feasibility/ safety)	NCT01032928	Single-arm, non-randomized trial	HNC; chronic dysphagia (> 12 months posttreatment) *N* = 30	In-clinic; RIP and nasal airflow biofeedback	Primary: Respiratory–swallow phase pattern Secondary: MBSImP, PAS, MDADI	Completed; published
Phase IIa (controlled efficacy)	NCT03377270	Randomized crossover trial	HNC; refractory dysphagia (> 6 months posttreatment) *N* = 40	In-clinic; RIP and nasal airflow biofeedback	Primary: MBSImP Secondary: PAS, FOIS, MDADI, PSS-HN	Completed; results summarized
Phase IIb (randomized efficacy)	NCT05278039	Randomized, sham-controlled, parallel-group trial	HNC; across recovery stages (≥ 3 months post-treatment) *N* = 88	Synchronous telehealth delivery; wearable sensor-based biofeedback; remote monitoring	Primary: Swallows initiated during expiration Secondary: PAS, NRRS, MBSImP, MDADI	Recruitment complete; data analysis ongoing

*Note.* HNC = head and neck cancer; RIP = respiratory inductance plethysmography; MBSImP = Modified Barium Swallow Impairment Profile (Martin-Harris et al., 2008); PAS = Penetration–Aspiration Scale; MDADI = MD Anderson Dysphagia Inventory (Chen et al., 2001); FOIS = Functional Oral Intake Scale (Crary et al., 2005); PSS-HN = Performance Status Scale for Head and Neck Cancer (List et al., 1996); NRRS = Normalized Residue Ratio Scale (Pearson et al., 2013).

In our Phase I single-arm, nonrandomized trial (NCT01032928), including individuals with chronic dysphagia following treatment for HNC (*n* = 30) who were more than 12 months post–cancer treatment (*M* = 3.4 years), we demonstrated that participants who received RST showed significant increases in optimal expiratory–swallow–expiratory (E-E) phase patterning, with the proportion of optimally timed swallows increasing from 43% preintervention to 86% postintervention (*p* < .0001). These changes were accompanied by significant improvements in key components of swallowing physiology, including reductions in impairment rates for laryngeal vestibular closure (from 78.2% to 55.5%, *p* = .0004), tongue base retraction (from 96.0% to 88.6%, *p* < .0001), and pharyngeal residue (from 96.1% to 88.4%, *p* = .01). Airway protection also showed significant improvement, with the proportion of swallows exhibiting penetration–aspiration (Penetration–Aspiration Scale ≥ 3) decreasing from 78.0% preintervention to 38.3% postintervention (*p* < .0001). Patient-reported swallowing-related quality of life, as measured by the MD Anderson Dysphagia Inventory, also improved modestly, reaching statistical but not clinical significance. Importantly, these physiologic gains were maintained at 1-month follow-up in the subset of participants (*n* = 15) who returned for reassessment, indicating at least short-term stability of the trained coordination pattern. Participants in this trial had long completed cancer treatment and traditional swallowing therapy yet exhibited persistent dysphagia, underscoring the capacity for coordination-based retraining even in the chronic phase (Martin-Harris et al., 2015).

Building on the feasibility and physiologic improvements observed in Phase I, our subsequent Phase IIa randomized crossover trial (NCT03377270) was designed to evaluate the efficacy of RST in a larger cohort of individuals with refractory dysphagia following treatment for HNC. Preliminary findings from this trial, which enrolled 40 participants more than 6 months post–cancer treatment (*M* = 2.4 years), indicate that increases in expiratory-phase swallow initiation are associated with more efficient airway closure, reduced vulnerability to airway invasion, and improvements in physiologic measures related to bolus clearance and overall swallow safety (see Table 3). Although RST is not designed to modify structural anatomy directly, restoring optimal timing appears to enhance the functional effectiveness of residual motor capacity across the aerodigestive tract, supporting respiratory–swallow coordination as a modifiable driver of swallowing safety and efficiency rather than a secondary correlate.

**Table 3.** Swallowing outcomes following respiratory–swallow training (NCT03377270).

Outcome	Baseline mean score	1-week post-RST mean score	*p* value	Score range
MBSImP pharyngeal total	11.50	10.17	< .001	0–32
Laryngeal elevation	0.86	0.75	< .001	0–3
Laryngeal vestibular closure	0.62	0.53	< .001	0–2
Pharyngeal stripping wave	0.76	0.66	< .001	0–2
Pharyngeal contraction	0.78	0.58	< .05	0–3
Pharyngoesophageal segment opening	0.74	0.67	< .05	0–3
Tongue base retraction	1.96	1.81	< .001	0–4
Max Penetration–Aspiration Scale	4.88	3.65	< .05	1–8

*Note.* Participants were randomized to immediate RST (Arm 1) or delayed RST (Arm 2); as no between-group differences were observed, results are reported with groups combined (*n* = 40). *p* values reflect within-subject change over time derived from longitudinal mixed-effects models accounting for repeated measures. Higher scores indicate greater impairment across all measures. RST = Respiratory–Swallow Training; MBSImP = Modified Barium Swallow Impairment Profile.

Notably, the benefits of RST demonstrated in individuals with chronic and refractory dysphagia following HNC (Graham et al., 2026; Martin-Harris, 2025; Martin-Harris et al., 2015) indicate that coordination-based training can yield meaningful gains even when traditional therapy approaches have been unsuccessful. Emerging data from our most recent Phase IIb randomized, sham-controlled, parallel-group trial (NCT05278039) suggest that RST may be feasible earlier in the recovery trajectory; however, evidence in subacute populations remains preliminary and requires further investigation.

Collectively, our clinical trial work supports the premise that respiratory–swallow coordination is a viable and impactful therapeutic target for improving swallowing in HNC-related dysphagia. By demonstrating consistent links between retrained coordination patterns and improved swallowing outcomes, this body of work provides a strong evidentiary foundation for integrating RST into comprehensive dysphagia rehabilitation programs and for extending evaluation beyond laboratory endpoints to real-world functional performance.

## Translation to Ambulatory Monitoring

The translation of respiratory–swallow coordination from laboratory-based assessment and training to ambulatory, real-world contexts represents a critical next step for both clinical implementation and research advancement and is currently being evaluated in our ongoing Phase IIb randomized, sham-controlled, parallel-group trial (NCT05278039) that incorporates telehealth delivery and ambulatory monitoring. Traditional, expensive, and nonportable instrumentation for the simultaneous acquisition of respiratory and swallowing data is inherently limited to controlled settings, constraining the ability to evaluate coordination during everyday eating and drinking (Hopkins-Rossabi et al., 2021; Rangwala et al., 2026). Recent advances in sensing, signal processing, and feedback delivery now enable continuous, unobtrusive monitoring of respiratory and swallowing biomechanics outside the clinic, allowing coordination to be measured, trained, and reinforced in the environments where dysphagia-related risk most often occurs (Kang et al., 2022).

Soft, skin-interfaced mechano-acoustic sensors and related wearable platforms support long-duration capture of respiratory and swallowing signals with sufficient temporal resolution to identify swallow onset, respiratory phase, and coordination patterns during routine activity (Kang et al., 2022). When integrated with automated detection and feedback methods developed for RST (Hopkins-Rossabi et al., 2020), these technologies provide the infrastructure needed for remote performance monitoring, adaptive feedback, and scalable delivery of coordination-based RST training. This approach shifts the focus from episodic, clinic-centered measurement to longitudinal characterization of coordination behavior across meals, days, and contexts. These capabilities form the foundation of our ongoing Phase IIb trial, which is evaluating telehealth-delivered RST supported by wearable sensing, remote monitoring, and ambulatory assessment.

Ambulatory monitoring also enables new analytic opportunities that are not feasible in laboratory paradigms. Repeated measures of respiratory–swallow timing can be used to quantify variability, stability, and generalization of trained behaviors, offering objective indices of learning and retention. These metrics may function as physiologic biomarkers, defined here as objective, quantifiable indicators of swallowing physiology, which can be used to track recovery trajectories, identify periods of elevated risk, and personalize training intensity and feedback schedules. Such data are particularly relevant for HNC populations, where swallowing performance may fluctuate with fatigue, bolus characteristics, and treatment-related comorbidities.

Our ongoing Phase IIb trial also provides an opportunity to evaluate these technologies within a clinically deployed telehealth model. From a translational perspective, remote monitoring and feedback platforms have the potential to expand access to RST dysphagia rehabilitation. Telehealth-enabled RST delivery may reduce barriers related to geography, mobility, and resource availability while maintaining fidelity to the underlying physiologic targets. Integration of ambulatory sensing with clinician oversight also supports hybrid care models in which in-clinic assessment establishes the coordination target and remote training reinforces skill acquisition and maintenance over time. Formal evaluation of implementation determinants, frameworks, and outcomes represents an important direction for future research.

## Future Directions and Conclusions

Future studies will be needed to define optimal thresholds, dosing, and outcome metrics for ambulatory respiratory–swallow coordination assessment and training and to leverage ambulatory data to identify real-world biomarkers that inform durability of treatment effects and guide retraining protocols. Key priorities include validating coordination-derived biomarkers, such as quantitative measures of respiratory–swallow timing, against established instrumental measures of swallowing impairment (e.g., Modified Barium Swallow Study–based physiologic indices); determining the durability and clinical significance of real-world coordination changes; establishing clinically and patient-identified meaningful thresholds for change; and evaluating implementation strategies to support broader clinical adoption of RST.

The current evidence supports respiratory–swallow coordination as a modifiable cross-system rehabilitation target linked to swallowing improvement in patients with refractory, chronic dysphagia following HNC treatment. Ongoing and future work will build on these digital health technologies and hybrid delivery models to extend our RST work to other populations, including HNC patients in more acute stages of recovery and patients with neurological disease (NCT04664634; Michael J. Fox Foundation, 022749).

## Data Availability Statement

Data sharing is not applicable to this article as no data sets were generated or analyzed during the current study.
